# Supplementary material for: More online interaction, more stock liquidity:——Evidence from Chinese stock exchange online interaction platform
Source: PLoS One. 2024 Sep 6;19(9):e0308160. doi: 10.1371/journal.pone.0308160 (PMC11379302; doi:10.1371/journal.pone.0308160)
Supplement: S1 Appendix — (DOCX) [file pone.0308160.s001.docx]

Appendix

Table A1 Different life cycles of enterprises

| Different life cycles of enterprises | | | | | | | | |
| --- | --- | --- | --- | --- | --- | --- | --- | --- |
|  | Growth period | | Mature period | Decline period | | | | |
| operate | - | + | + | - | + | + | - | - |
| invest | - | - | - | - | + | + | + | + |
| finance | + | + | - | - | + | - | + | - |

Note: This table presents the cycles of the enterprises.

Table A2 Main hypotheses and regression results

| **Hypothesis** | **Hypothetical content** | **Accept** |
| --- | --- | --- |
| **H1** | The online interaction has a positive effect on stock liquidity. | **Yes** |
| **H2** | The longer the expected tenure of executives, the significant the effect of the online interaction on stock liquidity. | **Yes** |
| **H3** | The higher the ratio of institutional ownership, the smaller the effect of the online interaction on stock liquidity. | **Yes** |
| **H4** | The impact of online interaction on stock liquidity is more significant for companies in the growth stage. | **Yes** |
| **H5** | The impact of online interaction on stock liquidity is more significant for non-state-owned enterprises. | **Yes** |
