# Supplementary material for: More online interaction, more stock liquidity:——Evidence from Chinese stock exchange online interaction platform
Source: PLoS One. 2024 Sep 6;19(9):e0308160. doi: 10.1371/journal.pone.0308160 (PMC11379302; doi:10.1371/journal.pone.0308160)
Supplement: Attachment — Submitted filename: Response to Reviewer Comments.docx [file pone.0308160.s002.docx]

Dear Editors and Reviewers:

Thank you for your letter and for the reviewers’ comments concerning our manuscript. Those comments are all valuable and very helpful for improving our paper, as well as the important guiding significance to our research. We have read comments carefully and have made revisions which we hope meets with approval. In what follows, we list each comment (in italics) and our response (in roman text). We also highlight the revised content in BLUE in the revised paper.

1. *Since you use a Chinese context, there are several recent papers on China’s institutional quality that need to be discussed. You can also design a few tests to examine the moderating effects of different institutional environment on the link between investor interaction and stock market liquidity, for example using formalism developed in An, J., Hou, W., & Zhang, Y. (2019). China’s rule of law in New Era: the rise of regulation and formalism. Journal of Chinese Economic and Business Studies, 17(3), 313-318, and bureaucrats checks developed in An, J., Armitage, S., Hou, W., & Liu, X. (2020). Do checks on bureaucrats improve firm value? Evidence from a natural experiment. Accounting & Finance, 60(5), 4821-4844.*

**Response**: This is a very meaningful suggestion. In order to examine the moderating effects of different institutional environment on the link between the online interaction and stock market liquidity, we referred to An, J., Armitage, S., Hou, W., & Liu, X. (2020), constructed two sets of measures on the extent to measure different institutional environment. The first set is SOE, which was a dummy variable that equals 1 if a firm’s controlling shareholder is a government entity, and 0 otherwise. Our second set of measures, State holding, was based on the percentages of firm shares owned by the state. The regression results show that the effect of online interaction on stock liquidity mainly affects non-state-owned enterprises. These results can be seen in our new Table 6.

**Position in the paper:** See section 4.3 “*Moderating effect analysis*”.

2. *The interaction you specifically looking at is facilitated by technology. I therefore suggest you motivate your paper incorporating a discussion of technology and finance. An, J., & Rau, R. (2021). Finance, technology and disruption. The European Journal of Finance, 27(4-5), 334-345, offers you some good background to start with. Similarly, An, J., Hou, W., & Lin, C. (2022). Epidemic disease and financial development. Journal of Financial Economics, 143(1), 332-358, also offers some hints on technology and finance.*

**Response:** Thank you for this valuable comment. I agree that the interaction I'm focusing on is significantly influenced by technology. I have incorporated a discussion on technology and finance in my paper, and added them to our literature review part.

*'***Position in the paper:** See section 2.1 “*Information technology and Stock market efficiency*”.

3. *Why the tables a showing up multiple times? Why do not add notes to the tables to make them self-contained. I suggest you follow the following studies to format your paper.*

**Response:** Thanks for your suggestions! We have added the notes and format the tables. See from Table 1 to Table 8.

4. *In your literature review, we suggest incorporating the following papers that are relevant to your study:
   - Bao, Z., & Huang, D. (2020). Gender differences in reaction to enforcement mechanisms: A large-scale natural field experiment.
   - Bao, Z., & Huang, D. (2021). Shadow banking in a crisis: Evidence from FinTech during COVID-19. Journal of Financial and Quantitative Analysis, 56(7), 2320–2355.
   - Chen, M., Li, N., Zheng, L., Huang, D., & Wu, B. (2022). Dynamic correlation of market connectivity, risk spillover and abnormal volatility in stock price. Physica A: Statistical Mechanics and Its Applications, 587, 126506.
   - Chen, M., Wang, Y., Wu, B., & Huang, D. (2021). Dynamic analyses of contagion risk and module evolution on the SSE a-shares market based on minimum information entropy. Entropy, 23(4), 434.
   Yu, D., & Huang, D. (2023a). Cross-sectional uncertainty and expected stock returns. Journal of Empirical Finance, 72, 321–340.
   Yu, D., & Huang, D. (2023b). Option-Implied Idiosyncratic Skewness and Expected Returns: Mind the Long Run. Available at SSRN 4323748.
   Yu, D., Huang, D., & Chen, L. (2023). Stock return predictability and cyclical movements in valuation ratios. Journal of Empirical Finance, 72, 36–53.
   These papers can provide valuable insights into the impact of information disclosure and interaction on market efficiency, as well as the role of social media in the capital market. For instance, Bao and Huang (2020) investigate the gender differences in reaction to enforcement mechanisms, which could be relevant to your study in understanding how different groups of investors may react to information interaction. Chen et al. (2022) explored the dynamic correlation of market connectivity, risk spillover, and abnormal volatility in stock price, which can help you better understand the relationship between information interaction and stock liquidity. By incorporating these papers into your literature review, you can provide a more comprehensive understanding of the existing research in this area and strengthen the theoretical foundation of your study. Yu, D.,& Huang, D. (2023a) highlighted the need for effective information management and the importance of understanding the impact of social media on market sentiment and efficiency, explored how different levels of cross-sectional uncertainty can impact the expected returns on stocks, proposing that higher uncertainty might lead to higher expected returns as a compensation for the increased risk.*

**Response:** Thanks for your suggestions! We have read the existing literature carefully. In terms of the relationship between the information technology and market efficiency, most existing literature shows that the information technology can improve the information disclosure, the stock market efficiency. Bao, Z., and Huang, D. (2021) explored that the rapid development of technology had given rise to fintech platforms, which provided an important venue for investors to discuss, analyze, and disseminate their views on market trends, risks, and opportunities. As a kind of social media, it could not only improve the timeliness of data analysis and sharing, but also improved the information transparency of financial institutions, promoted better risk management and improved the pricing efficiency of capital market. Chen, M., Li, N., Zheng, L., Huang, D., & Wu, B. (2022) explored how information shared across different market participants, facilitated by technological advancements and social media, contributed to market connectivity. This connectivity, while beneficial for the flow of information, also leaded to risk spillover, where the risk in one market segment could quickly affect others, contributing to abnormal stock price volatility. Chen, M., Wang, Y., Wu, B., & Huang, D. (2021) used the concept of minimum information entropy, highlighted the importance of efficient and transparent information disclosure in mitigating contagion risks particularly in the age of social media. Yu, D., & Huang, D. (2023b), found information was rapidly disseminated through various channels including social media, such market sentiments could be quickly amplified, impacting stock prices and investor behavior. Yu, D., Huang, D., & Chen, L. (2023) argued that better information disclosure and effective interaction among market participants leaded to more accurate and efficient pricing, as reflected in these valuation ratios.

**Position in the paper:** See section 2.1 “Information technology and Stock market efficiency”.

5. *Please provide a more detailed explanation of the methodology used in your study, including the specific statistical tests and models employed. This will help readers better understand your approach and assess the validity of your findings.*

**Response:** Thank you for this valuable comment. In response, we have enriched our manuscript with a more detailed explanation of the methodology. This includes a thorough elucidation of the variable selection, various statistical tests and analytical models we utilized. This enhanced methodological exposition aims to provide a clearer and more robust framework for understanding the scope and rigor of our analytical processes.

**Position in the paper:** See section 3 “Sample selection model development and variable definitions ”.

6. *Consider discussing the potential limitations of your study, such as the generalizability of your findings to other markets or the potential biases in the data collected from the interaction platforms.*

**Response:** Thank you for this valuable comment. Like any study, ours is not without limitations. Firstly, the application of this paper’s conclusions to other countries is not tested in the current study. The prior research detected an intense interconnection between the major social media cross countries. This raises the possibility that our findings, while derived from a Chinese context, may have broader applicability to online platforms in other countries. However, due to the limitation of data availability and article space, we have not verified these within the scope of our current research. Secondly, the methodological approach employed in this study utilized relatively straightforward measurement indicators. While these indicators provided a foundational understanding, they may lack the depth and sophistication achievable with more advanced analytical tools and methods. Lastly, in the future, we could develop a more comprehensive set of metrics, explored which type of interaction was most effective in conveying valuable information. This presented both a fascinating and substantial opportunity for further research.

**Position in the paper:** See section 9 “Limitations and future research directions”.

7. *It would be helpful to include a more in-depth discussion of the practical implications of your findings for both investors and firms. This can help readers better understand the real-world significance of your research.*

**Response:** This is a very meaningful suggestion. First, the findings of this study guide enterprises to pay attention to the important role of social media on its stock liquidity. Since, the traditional one-way information transmission method can no longer meet the needs of investors. Enterprises should pay attention to the dynamics of investor information on social media represented by interactive platforms, understand the issues that investors care about, and respond to investors' questions in a timely and proactive manner. Strengthen the interactive communication with investors, improve the external information environment of enterprises, and reduce the information asymmetry between investors and enterprises.

Second, the heterogeneity test of this paper finds that the interaction between network platforms and stock liquidity has a certain degree of heterogeneity. Private enterprises and enterprises in growth stage should pay more attention to set up an efficient interactive platform to provide investors with more accurate and targeted information, which help to maintain the efficiency of corporate capital market information.

Third, this paper finds that sometimes “noise” is generated in the process of interaction. Some enterprises’ reply is unnecessarily too long, without information, and poor response timeliness. Even more, some executives use the online interactive platform to manipulate information disclosure out of self-interest. It is necessary to standardize the management of interactive platforms. The supervision department should actively guide enterprises to improve the quality of information disclosure, and meantime conduct strict supervision on underperforming enterprises.

**Position in the paper:** See section 8 “Practical implications”.

8. *Other revisions:*

**Abstract changed:** We think the original abstract are a bit wordy. In order to make the abstract easier to read, we slightly change it.

We tried our best to improve the manuscript and made some minor changes in the manuscript. These changes will not influence the content and framework of the paper. We appreciate for Editors/Reviewers’ warm work earnestly, and hope that the correction will meet with approval.

Once again, thank you very much for your comments and suggestions.

**References**

An, J., Hou, W., & Zhang, Y. (2019). China’s rule of law in New Era: the rise of regulation and formalism. Journal of Chinese Economic and Business Studies, 17(3), 313-318

An, J., Armitage, S., Hou, W., & Liu, X. (2020). Do checks on bureaucrats improve firm value? Evidence from a natural experiment. Accounting & Finance, 60(5), 4821-4844.

An, J., & Rau, R. (2021). Finance, technology and disruption. The European Journal of Finance, 27(4-5), 334-345

 An, J., Hou, W., & Lin, C. (2022). Epidemic disease and financial development. Journal of Financial Economics, 143(1), 332-358

Bao, Z., & Huang, D. (2020). Gender differences in reaction to enforcement mechanisms: A large-scale natural field experiment.

Bao, Z., & Huang, D. (2021). Shadow banking in a crisis: Evidence from FinTech during COVID-19. Journal of Financial and Quantitative Analysis, 56(7), 2320–2355.

Chen, M., Li, N., Zheng, L., Huang, D., & Wu, B. (2022). Dynamic correlation of market connectivity, risk spillover and abnormal volatility in stock price. Physica A: Statistical Mechanics and Its Applications, 587, 126506.

Chen, M., Wang, Y., Wu, B., & Huang, D. (2021). Dynamic analyses of contagion risk and module evolution on the SSE a-shares market based on minimum information entropy. Entropy, 23(4), 434.

Yu, D., & Huang, D. (2023a). Cross-sectional uncertainty and expected stock returns. Journal of Empirical Finance, 72, 321–340.

Yu, D., & Huang, D. (2023b). Option-Implied Idiosyncratic Skewness and Expected Returns: Mind the Long Run. Available at SSRN 4323748.

Yu, D., Huang, D., & Chen, L. (2023). Stock return predictability and cyclical movements in valuation ratios. Journal of Empirical Finance, 72, 36–53.
