# Supplementary material for: More online interaction, more stock liquidity:——Evidence from Chinese stock exchange online interaction platform
Source: PLoS One. 2024 Sep 6;19(9):e0308160. doi: 10.1371/journal.pone.0308160 (PMC11379302; doi:10.1371/journal.pone.0308160)
Supplement: Attachment — Submitted filename: Response to Reviewer Comments.docx [file pone.0308160.s003.docx]

Dear Editors and Reviewers:

Thank you for your letter and for the reviewers’ comments concerning our manuscript. Those comments are all valuable and very helpful for improving our paper, as well as the important guiding significance to our research. We have read comments carefully and have made revisions which we hope meets with approval. In what follows, we list each comment (in italics) and our response (in roman text). We also highlight the revised content in BLUE in the revised paper.

1. *The language of newly added Section 2.1 should be re-organized in a logic way.*

**Response**: This is a very meaningful suggestion. We appreciated your feedback on Section 2.1. We have restructured it to ensure that the ideas are presented more logically and coherently. Thank you for pointing this out.

**Position in the paper:** See section 2.1 “Information technology and Stock market efficiency”.

2. *The presentation of tables (such as table 5) should be improved. For instance, the sequence of rows of controls seems mixed.*

**Response:** Thanks for your suggestions! We have revisited the table and reorganized the sequence of the control rows to enhance clarity and coherence.

**Position in the paper:** See from Table1 to Table 8.

3. *Section7 (the contribution section) lacks proper citations of existing literature.*

**Response:** Thank you for this valuable comment. In response, we have thoroughly reviewed this section and incorporated appropriate citations to ensure our contributions are well-supported.

**Position in the paper:** See section 6.1 “*Theoretical implications*”.

4. *Please also discuss endogeneity issues in section 9 and suggest possible solutions, moreover, the potential biases in the data collected from the interaction platforms (as mentioned by last report) haven’t been discussed.*

**Response:** Thank you for this valuable comment. In this current version，we have discussed the endogeneity problems and limitations of this paper, and discuss the potential bias that We may have in data collection. We propose feasible solutions to alleviate these problems based on the methods of replacing explanatory variables and explain variables in the DID model. Your feedback helps to improve the rigor and comprehensiveness of our research.

**Position in the paper:** See section 6.4 “*Limitations and future research directions*”.

5. *Sections 6-10 can be put together for better readability.*

**Response:** Thank you for the suggestion. We have restructured these sections and put them into one section. Consolidating these sections may indeed provide a more cohesive and comprehensive narrative.

**Position in the paper:** See section 6 “*Conclusions”.*

We tried our best to improve the manuscript and made some minor changes in the manuscript. These changes will not influence the content and framework of the paper. We appreciate for Editors/Reviewers’ warm work earnestly, and hope that the correction will meet with approval.

Once again, thank you very much for your comments and suggestions.
