# Supplementary material for: More online interaction, more stock liquidity:——Evidence from Chinese stock exchange online interaction platform
Source: PLoS One. 2024 Sep 6;19(9):e0308160. doi: 10.1371/journal.pone.0308160 (PMC11379302; doi:10.1371/journal.pone.0308160)
Supplement: Attachment — Submitted filename: Response to Reviewer Comments.docx [file pone.0308160.s004.docx]

Dear Editors and Reviewers:

Thank you for your letter and for the reviewers’ comments concerning our manuscript. Those comments are all valuable and very helpful for improving our paper, as well as the important guiding significance to our research. We have read comments carefully and have made revisions which we hope meets with approval. In what follows, we list each comment (in italics) and our response (in roman text). We also highlight the revised content in BLUE in the revised paper.

1. *Overall, this revision largely improves the quality of this paper. I suggest that autghor should check the typos/formatting problem in 6.1. Theoretical implications.*

**Response**: Thank you for recognizing the improvements in our paper. We have carefully reviewed and corrected the typos and formatting issues in section 6.1 Theoretical Implications. We appreciate your valuable feedback!

**Position in the paper:** See section 6.1 “Theoretical implications”.

2. *The authors adequately address the issues raised by the previous reviewer. However, these are a few issues when addressed by the authors will help the readers understand and appreciate the concept. These issues are stated below:The abstract did not show what the problem authors want to solve, the approach they intend to delve into the problem, and how significant it is in investigating and solving the problem. The abstract gives adequate information to the reader about the research and its conclusion.It is difficult to tell whether these are the research findings or study objectives being presented in the introduction. These make the paper complex for readers. If they represent gaps found in the literature, they should be properly cited, or if they are study objectives, they should be well stated.The paper lacks clarity and has repetitive sentences. The paper needs a thorough reading to correct minor grammatical errors. Besides, research is about facts, but the authors say “We believe that as the managers of a company…” It sounds accurate if the authors can reframe the sentences.The results of the hypothesis can be summarized by showing the accepted and rejected results in a table to enable readers to understand better.*

**Response:** Thanks for your valuable suggestions. Firstly, we revised the abstract to clearly state the problem we try to investigate and our research findings. Secondly, in the introduction, we rewrote research gaps, findings and contributions to enable readers to understand better. Finally, in order to make it easier for readers to read and understand this paper, we presented the main hypotheses and results of this paper in Appendix A2.

**Position in the paper:** See abstract, section 1 “Introduction” and Appendix A2.

We tried our best to improve the manuscript and made some minor changes in the manuscript. These changes will not influence the content and framework of the paper. We appreciate for Editors/Reviewers’ warm work earnestly, and hope that the correction will meet with approval.

Once again, thank you very much for your comments and suggestions.
